# Increasing Antioxidant Activity and Protein Digestibility in *Phaseolus vulgaris* and *Avena sativa* by Fermentation with the *Pleurotus ostreatus* Fungus

**DOI:** 10.3390/molecules22122275

**Published:** 2017-12-20

**Authors:** Edith Espinosa-Páez, Ma. Guadalupe Alanis-Guzmán, Carlos E. Hernández-Luna, Juan G. Báez-González, Carlos A. Amaya-Guerra, Ana M. Andrés-Grau

**Affiliations:** 1 Facultad de Ciencias Biológicas, Universidad Autónoma de Nuevo León, Ave. Universidad s/n, Cd. Universitaria, 66450 San Nicolás de los Garza, Mexico; edith.espinosapz@uanl.edu.mx (E.E.-P.); maria.alanisgm@uanl.edu.mx (M.G.A.-G.); carlosehlmx@yahoo.com (C.E.H.-L); numisamaya@hotmail.com (C.A.A.-G.); 2 Instituto Universitario de Ingeniería de Alimentos para el Desarrollo, Universitat Politècnica de València, Camino de Vera s/n, 46022 Valencia, Spain; aandres@tal.upv.es

**Keywords:** *Pleurotus ostreatus*, antioxidant activity, polyphenols, digestibility, fermentation, cereals, legumes

## Abstract

The aim of the research was to determine the impact of fermentation with *Pleurotus ostreatus* on kidney beans, black beans, and oats. The results indicate that the fungus has a positive effect on the substrates when compared to the controls. The antioxidant activity (39.5% on kidney beans and 225% on oats in relation to the controls) and content of total polyphenols (kidney beans three times higher regarding the controls) increased significantly by the presence of the fungus mycelium, even after simulated digestion. There was a significant increase in protein digestibility (from 39.99 to 48.13% in black beans, 44.06 to 69.01% in kidney beans, and 63.25 to 70.01% in oats) and a decrease of antinutrient tannins (from 65.21 to 22.07 mg in black beans, 35.54 to 23.37 in kidney beans, and 55.67 to 28.11 in oats) as well as an increase in the contents of some essential amino acids. Overall, this fermentation treatment with *Pleurotus ostreatus* improved the nutritional quality of cereals and legumes, making them potential ingredients for the elaboration and/or fortification of foods for human nutrition.

## 1. Introduction

Foods today are intended not only to satisfy hunger and provide the necessary nutrients for humans but also to prevent nutrition-related diseases that impact physical and mental wellness [1]. Functional foods have been introduced in markets, and they are usually defined as “modified foods which contain ingredients that have demonstrated actions that increase the welfare of the individual or decrease disease risk beyond the traditional role” [2]. The legume, a particularly common bean (*Phaseolus vulgaris*), is one of the main sources of vegetable protein available in developing countries [2]. The high lysine content protein of *Phaseolus vulgaris* makes it an ideal cereal protein; it supplements the deficiency in this essential amino acid and is also a staple ingredient in developing countries, where the availability of animal protein is low. Also, it provides adequate nutrition due to its contribution of carbohydrates [3] and high-quality protein. *Phaseolus vulgaris* has also been associated with various health benefits, including the reduced risk of diabetes and cardiovascular disease attributed to the presence of polyphenols [4,5]. *Phaseolus vulgaris*, however, contains antinutritional factors such as protein inhibitors (inhibitors of trypsin, chymotrypsin, and amylase), lectins, phytates, and tannins [6].

The common oat (*Avena sativa*) is among the major cereals used for human food [7]; it is the cereal with the highest percentage of vegetable fat and has a variety of minerals, trace elements and vitamins such as calcium, copper, iron, magnesium, potassium, selenium, zinc and vitamins: B1, B2, B3, B6 and E, and trace amounts of vitamin D [8].

The disadvantage of using legumes in food fortification is the presence of antinutrients such as phytates, tannins, and trypsin inhibitors, which decrease the digestibility of the protein [7].

Fungi are considered a source of food with incalculable value for their nutritional quality, being low in calories and rich in carbohydrates, essential amino acids, fiber, vitamins, and minerals [9,10]. Studies indicate that *Pleurotus* species are potent biological agents that convert non-food organic products into palatable human food [11]. They are able to synthesize a greater proportion of essential amino acids, which promotes a positive balance of amino acids, as well as improving the taste. They can grow on a variety of substrates, such as straw (wheat, oats, and rice), sawdust, cotton waste, banana leaves, corn stalks, and other agricultural wastes [12].

*Pleurotus ostreatus* is the second most cultivated edible mushroom worldwide after *Agaricus bisporus;* it has a high nutritional value as it contains minerals, vitamins, and proteins. While it has a low content of fat and sodium, it is high in potassium [10]. This fungus also has antioxidant properties [13,14].

The production of fermented foods is one of the oldest food processing technologies, [15] and it is an economic and simple process that causes chemical changes and modifies the functionality of foods [16]. Many of these foods are manufactured for their unique flavor, aroma, and texture attributes that are highly appreciated by the consumer. Furthermore, filamentous fungi simultaneously decrease anti-nutrients components and partially hydrolyzed biopolymers substrates. The byproduct of the fermentation can be used as an inexpensive food and as a supplement to support marketing demands [15]. Considering the above and the growing industry of ingredients and functional foods, the aim of this research was to evaluate the effect of fermentation with *Pleurotus ostreatus* on protein digestibility, antioxidant activity, and nutritional quality of cereals (oats) and legumes (black and kidney beans).

## 2. Results and Discussion

The obtained amount of flour, including mycelium produced during fermentation with *Pleurotus ostreatus*, is equivalent to the grams of substrate used in dry weight (BB, KB, OG), so there is only one bioconversion of the substrate to mycelium.

### 2.1. Proximal Chemical Analysis

The results of the chemical composition of dry matter corresponding to the non-fermented varieties of black beans, kidney beans and oats (BB, KB, OG) and fermented ones with *Pleurotus ostreatus* (FBB, FKB, FOG) are shown in Table 1. The effect of fermentation with a significant increase of 13% and 6% in kidney beans and oats protein, respectively, can be observed. This is attributed to the increase of amino acid synthesis as a consequence of the fermentation with *Pleurotus ostreatus* [12]. With respect to the dietary fiber content, values obtained for the legumes were as follows: 45.09 g (BB), 27.80 g (KB) and 13.48 g (OG), which are greater than those reported by the USDA, which are 5.5 g for raw black beans, 8.7 g for cooked black beans, 4.9 g for raw kidney beans, 9.3 g for cooked kidney beans, and 10.6 g for raw oats and 2.6 g for cooked oats [17]. In the fermented black beans and oats, the dietary fiber contents significantly decreased by 59% and 22% respectively, attributable to the action of the enzymes from *Pleurotus ostreatus* such as cellulase, hemicellulase, xynalases and laccases [18], which selectively use the lignin and cellulose for their growth; lignin and cellulose form the major composite of the dietary fiber in legumes and cereals. The decrease in these structural carbohydrates allows the transformation of resistant starch into available starch, which in the case of black beans is a cause of the high observed value of dietary fiber (48.73%). In contrast to FKB, the fiber significantly increased 16%. These differences could be explained, as the action of the fungus depends on the substrate, species, or the variety of substrate being used; the fungus adjusts its enzymatic systems (hydrolase enzymes, oxide reductases, etc.) in relation to the conditions of the substrate, mainly the presence of carbon and nitrogen, selective delignification, crude protein content, dry matter and the threshold availability of the substrate in the grain for the growth of the fungus after inoculation [19]. The way in which enzymes of the fungus act to obtain the nutrients necessary for growth depends on the substrate; in the case of FB, it has a higher hardness index [20], having less permeability so the fungus could not act the same way as in the black beans, which could explain the differences in the composition [21]. This hypothesis is confirmed by data shown in Table 2; in the case of unfermented and fermented kidney beans, they do not show an increase in the total polyphenol content and antioxidant activity. The fat content highlights a significant increase of 97% in the FOG regarding OG treatment, which may have been provided by the fungus fat, since a 4.8% value of this component in *Pleurotus ostreatus* was reported [22]; this also happened to the legumes, assuming that the fungus is found in a greater proportion in oats, due to the fact that cereals such as grains of beer, wheat, rice, oats, and corn are more accessible substrates for the fungus, thus growing easier on them [23], as observed with other species of this fungus such as *Pleurotus pulmonaris,* which report a 3% value of this component [18]. It is important to consider that increases in some nutrients may be a response to the percentage decrease in others.

### 2.2. Antioxidant Activity and Total Phenols

The content of total phenolic and antioxidant activity in flours was assessed before and after simulated gastric and intestinal digestion. The presence of total phenols in legumes and cereals has been documented in previous studies [5]. The initial content of phenols for the different treatments (Table 2) was 0.85 to 2.89 mg of gallic acid/g of flour, while Zielin´ski and Kozłowska [24] obtained 2.89 mg/g for FOG as the highest. Treatments of black beans and oats with *Pleurotus ostreatus* had a significant increase in the total phenol contents, i.e., 26.35% on BB and 240% in relation to the OG. This fungus is basidiomycete, which excreted at least three different oxidases of phenol; these are used to degrade lignin and obtain carbon and other nutrients. These laccases (phenol oxidases) are independent agents that catalyze reactions, including the oxidation of Mn^+2^ and Fe^+2^, that can polymerize, depolymerize or transform a wide range of phenolic compounds [25].

This increase may be due not only to the synthesis of phenols in the mycelium or hydrolysis of conjugated phenolics [26], but also to the deamination of aromatic amino acids phenylalanine and tyrosine precursor of phenolic acids [27]; in addition, the phenol oxidases of the fungus also produced interesting industrial bioconversions of many aromatic xenobiotic compounds from lignin [28]. The antioxidant activity in the treatments was 1.2 to 1.73 mg Trolox equivalent/g in legume flours—higher than the values obtained for oats 0.40 mg/g OG and 1.30 mg/g FOG; this variation between oat and bean flours may be due simply to the different types of antioxidant compounds that they contain, as well as to their concentration [29,30]. In relation to the effect of the fungus in the flour, the fermented black bean and oat treatments significantly increased antioxidant activity; FBB presented an increase of 39.5% on BB and FOG 225% in relation to the OG, attributing it to the fungus in these treatments. There was a significant increase of polyphenols due to depolymerization or hydrolysis of conjugated polyphenols, not occurring in FKB, in which there was no antioxidant activity increase; assuming that the threshold availability of the compounds is not the same due the fact that the kidney bean hull is harder [20] because it has a higher cellulose content than the black bean hull [31]. The fungus degrades lignin molecules by the action of ligninolytic enzymes (lignin peroxidase, manganese peroxidase and laccase) and can then access energy-rich polysaccharides for growth and metabolism [32]. In this case, the phenol oxidase enzymes could not act in the same way, degrading conjugated phenolic compounds by not having enough access to them [33]. The varieties of beans have different characteristics. Depending on the natural adaptation to the environment and the harder seed coat (hull), the permeability and the possibility of access of microorganisms decrease, so the hull protects the endosperm from microbial attacks, assuming greater resistance to the action of the fungus [21]. After digestion, the antioxidant activity increased both in non-fermented flour and fermented flour, reaching values of Trolox 3.04 to 8.97 mg/g and 5.04 to 13.31 mg/g, respectively. This presented a seven-fold increase in FBB.

Concerning the activities of the total phenolic content in simulated digestion, it was observed that at the end of the digestion, values ranged from 2.72 to 4.23 mg/g in non-fermented and from 4.91 to 6.85 mg/g in fermented flours with the fungus, increasing three-fold in FBB. This increase was the tendency in all treatments. This is contrary to the results published by other authors [34], reporting that the content of total phenolic and antioxidant activity tended to decrease after digestion, due to the low pH fluids during gastric digestion and the interaction with other compounds such as minerals, fiber, and protein in foods, affecting the solubility and availability of polyphenols. However, it is well known that anthocyanins (antioxidant compounds present in many legumes), resist low pH and protect themselves; this probably explains the tendency observed in our results. In addition, we attribute it to the action of the enzymes of the fungus in the fiber, leaving it more available or accessible to antioxidants. This is very important since antioxidants play a protective role in the gastrointestinal tract while keeping the redox balance against harmful antioxidant agents, helping the prevention of gastrointestinal diseases during the process of digestion [34].

### 2.3. In Vitro Digestibility, Soluble Nitrogen and Tannins

In vitro digestibility values obtained for non-fermented bean flours shown in Table 3, were BB 39.99% and KB 44.06%. These were higher than those reported previously [35], which reported values below 35% in *Phaseolus vulgaris* in raw and pre-cooked flours. Fermented black beans with the fungus (FBB) presented a digestibility of 48.13%, which is similar to values reported for black beans fermented with *Bacillus* sp. [36]. The kidney bean with *Pleurotus ostreatus* (FKB) had a higher digestibility with 69%, and is higher than other reports [35], which have values below 50% for raw and cooked carica beans. Similarly, both treatments of fermented beans (FBB and FKB) had a higher digestibility than those reported for beans fermented with other microorganisms, such as *Rhizopus microsporus* var., *Chinensis* and *Lactobacillus plantarum* 33.87 and 35.09%, respectively [37]. The digestibility values for OG and FOG flours were 63.25% and 70% respectively, being similar to those reported by other authors [38]. The protein digestibility increased significantly in all fermented with *Pleurotus ostreatus* because this fungus has a great selectivity of delignification, which degrades the substrate and makes proteins more digestible [39].

Soluble nitrogen values presented a significant difference between fermented and non-fermented treatments with *Pleurotus ostreatus*, confirming the effect of *Pleurotus ostreatus* on the availability of the protein (Table 3). In addition, the ability of the fungus to reduce tannins favored the increase in protein digestibility [40]. Tannin contents presented in Table 3 show a significant decrease in all fermented products with 66% for FBB, 34% for FKB and 49% for FOG. Fan et al. [41] reported that the fungus is able to reduce or eliminate tannin antinutrients mainly by the action of a tannase present in the fungus, which ultimately destroys the tannins [42]. The tannin values of non-fermented beans samples were similar to those previously reported [43]. The decrease in the concentration of tannins has been reported in the lactic fermentation of *Phaseolusvulgaris* [27].

### 2.4. Amino Acid Profile

The results of the amino acid profiles are shown in Table 4. It is observed that in oats, as well as in fermented legumes, a significant increase of most of the essential amino acids (isoleucine, leucine, phenylalanine, valine, threonine, and methionine) is present, confirming the effect of *Pleurotus ostreatus* in the synthesis of essential amino acids [12]. This also highlights a significant increase in sulfur amino acids such as methionine and cysteine with values of 22.4 mg/g of protein and 49.19 mg/g of protein in fermented treatments of kidney beans and oats respectively; this is considered relevant to improve the quality of the protein in the flour. Basic amino acids such as lysine and arginine decreased in all treatments in the fermentation process with the fungus. These amino acids were probably destabilized by the acidic conditions associated with the fermentation since the process was maintained at pH < 4 [44,45]. The values of these amino acids in the fermented products are similar to those reported for *Pleurotus ostreatus* [12].

## 3. Materials and Methods

### 3.1. Seeds, Microorganism and Maintenance

The black beans (BB), kidney beans (KB) and oats grain (OG) were obtained from the local food market in Gpe, N. L. Mexico. *Pleurotus ostreatus* CS155 strain was obtained from Laboratorio de Enzimología, Facultad de Ciencias Biológicas, Universidad Autónoma de Nuevo León, San Nicolas de los Garza, N.L., Mexico. This strain was maintained by periodic transfers (2–3 months) in Petri dishes with growth medium prepared with 0.4% yeast extract, 0.1% malt extract, 0.4% glucose and 1.5% agar (YMGA) [46].

### 3.2. Inoculum Production

Seeds of fresh black beans, kidney beans and oats were obtained from local suppliers; they were washed and sterilized in mason jars with autoclave 121 °C for 45 min and water rational of 1:1, 1:1.10, and 1:1.35 (*w*:*v*), respectively. The strain fungus was inoculated in a YMG medium (0.44% yeast extract, 0.1% malt extract and 0.4% glucose) and incubated under agitation (150 rpm) for two weeks at room temperature, based on the studies by Hernandez, et al., 2008 and Gan, et al., 2017 [46,47]. The culture was homogenized during four periods of 15 s and used as inoculum. Then, 8 mL of the homogenized culture that contains 2.64 mg of biomass [d.w.] per gram of the homogenized culture [48] was added to each pre-treated jar for solid fermentation, looking for the inoculum to cover the whole sample while affecting the ratio of nutrients as little as possible. Afterwards, looking for sufficient biomass, the jars with the inoculated substrates were incubated for 2 weeks at room temperature under agitated anaerobic conditions. Subsequently, at the beginning of the idiophase, the highest ratio of biomass to the volume of medium nutrient-limited liquid was obtained, according to preliminary tests performed in our laboratory, where we observed that in a period of two weeks, the seeds were 100% colonized. The grains with mycelium were ground (Moulinex, Écully, France) and dehydrated in a convection furnace at 70 °C. Flours obtained with fermented and unfermented grains were labeled as black bean (BB); black bean with *Pleurotus ostreatus* (FBB); kidney beans (KB); kidney bean with *Pleurotus ostreatus* (FKB); oats (OG) and oats with *Pleurotus ostreatus* (FOG) [46].

### 3.3. Proximal Chemical Analysis

All proximal analyses were performed using standard methods of Association of Official Analytical Chemistry [49]. Protein content was determined with the Kjeldahl method (AOAC 930.29). Fat content was measured using the Goldfish method (AOAC 920.36C). Ash content was evaluated gravimetrically (AOAC 14.006), and dietary fiber and available carbohydrates were measured with the gravimetric-enzymatic (AOAC 985.29) and chemical (AOAC 962.09) methods, respectively. 

### 3.4. Simulated In Vitro Digestion

A protocol based on the use of digestive enzymes was followed [50] and simulated fluids were prepared according to the protocol proposed by Minekus et al. (2014) [51]. Portions of 5 g of each sample were placed into a 50-mL tube. For the oral phase, 5 mL of FOS (simulated oral fluid) was added, incubated for 5 min at 37 °C in agitation. Then, 12 mL of FGS (simulated gastric fluid) with pepsin at pH 2.3 was added following incubation for 2 h at 37 °C in 55 rpm orbital agitation for the gastric phase. Finally, 20 mL FIS (simulated intestinal fluid) with 1.98 mg of pancreatin and bile extract at pH 8 was added and incubated for 2 h at 37 °C on orbital agitation for the intestinal phase.

### 3.5. Antioxidant Activity 

Antioxidant activity was measured in samples before and after in vitro digestion. For undigested samples, the determination was made with extractions, using methanol 80% 1:5 (*p*:*v*) for each sample. The determinations of the extractions after gastric and intestinal digestion were performed with methanol 80% 1:20 (*v*:*v*) from 4 mL of the product of the gastric phase and 8 mL of the product of the intestinal phase. Of each extraction, 0.1 mL was mixed with 3.9 mL of DPPH (1 N), incubated for 30 min in darkness and measured at 515 nm absorbance. The results were expressed in the mg equivalent of Trolox [52].

### 3.6. Total Phenol Content 

Total phenol content was determined in all samples before and after in vitro digestion. All the sample extractions were made with methanol 80% 1:5 (*w*:*v*) for undigested samples. For determination after gastric and intestinal digestion, extractions were performed with methanol 80% 1:20 (*v*:*v*) from 4 mL of the product of the gastric phase and 8 mL of the product of the intestinal phase. A 1 mL of extract was mixed with 0.025 mL of Folin–Ciocalteu (1 N), 2.5 mL of sodium carbonate (20%), incubated for 40 min in darkness, and then measured at 725 nm absorbance. The results were expressed as equivalents of gallic acid [52].

### 3.7. Protein Digestibility

A simulated digestion was performed and 37 mL of intestinal phase product was obtained; protein not digested was precipitated with trichloroacetic acid (TCA) and was prepared to a final concentration of 12% (*w*/*w*). It was centrifuged at 3500 rpm for 15 min and was then decanted. The precipitate was washed and centrifuged twice, and its nitrogen content was determined by the Kjeldahl [51,53].

### 3.8. Soluble Nitrogen 

For soluble nitrogen, a 0.15-g sample was placed into a 50-mL tube, to which 49.5 mL of NaOH 0.02 N was added; it was then stirred for 1 h and centrifuged for 5 min at 3000 rpm. The content of nitrogen in the supernatant was determined by micro-Kjeldahl [54].

### 3.9. Tannins Content

Tannin content was measured using the AOAC 952.03 method (AOAC 1990). The standard curve was prepared with 100, 200, 400, 600 and 800 aliquots in 1000 µL of a tannic acid stock solution of 0.1 mg/mL. Each 10 mg sample was dissolved in 10 mL of water and 1 mL was taken to make the determinations. We then added 7.5 mL of water, 500 µL of Folin–Deniss and 1 mL of Na_2_CO_3_ at 35%; 10 mL was taken and stirred. After 30 min, a spectrophotometer at 760 nm absorbance was used to measure results. The results are expressed as equivalents of tannic acid [49].

### 3.10. Amino Acids Profile

The amino acid profile was determined using high-performance liquid chromatography (HPLC), gas-liquid chromatography (GLC) and mass spectrometry (MS), according to the method AOAC 982.30 E (a,b,c) [49].

### 3.11. Statistical Analysis

Data from the three replicated experiments were analyzed to determine whether the variances were statistically homogeneous, and the results expressed as means ± SD. Statistical comparisons were made by one-way analysis of variance (ANOVA) followed by a Duncan´s test using SPSS 17 Software. Difference between means were considered significant at *p* < 0.05.

## 4. Conclusions

*Pleurotus ostreatus* has a positive effect on the two varieties of beans and oats, increasing the content of polyphenols and their antioxidant activity even during digestion, thus improving the digestibility of the protein and decreasing tannins. The impact on the content of amino acids shows an increase of sulfur amino acids promoted by the fermentation of legumes and cereal, increasing the potential of these flours as functional ingredients in the production of food for human nutrition.

## Figures and Tables

**Table 1:** Nutritional components of different obtained flours.

Parameter (%)	BB	FBB	KB	FKB	OG	FOG
Protein	23.62 ± 1.12 ^c^	22.80 ± 2.85 ^c^	22.81 ± 1.3 ^c^	25.78 ± 2.35 ^d^	11.78 ± 1.28 ^a^	12.56 ± 0.63 ^b^
Fat	2.08 ± 0.32 ^bc^	1.67 ± 0.34 ^abc^	1.86 ± 0.66 ^ab^	1.68 ± 0.17 ^a^	2.44 ± 0.45 ^c^	4.80 ± 0.60 ^d^
Minerals	4.90 ± 0.15 ^c^	4.58 ± 0.31 ^c^	4.63 ± 0.24 ^c^	4.02 ± 0.01 ^c^	1.61 ± 0.09 ^b^	0.83 ± 0.52 ^a^
Fiber	48.73 ± 0.56 ^f^	20.0 ± 0.51 ^c^	29.18 ± 0.15 ^d^	33.88 ± 0.28 ^e^	14.00 ± 0.07 ^b^	10.92 ± 0.70 ^a^
Carbohydrates	20.69 ± 0.79 ^a^	50.95 ± 0.96 ^d^	41.52 ± 0.59 ^c^	34.64 ± 1.5 ^b^	70.17 ± 0.44 ^e^	70.89 ± 0.66 ^f^

Black beans (BB); Black beans with *Pleurotus ostreatus* (FBB); kidney beans (KB); kidney bean with *Pleurotus ostreatus* (FKB); oats (OG) and oats with *Pleurotus ostreatus* (FOG). Average values with three replicates ± standard deviations, of three different lots. Mean values labeled with a different letter in the same file are significantly different (*p* < 0.05).

**Table 2:** Effect of *Pleurotus ostreatus* on antioxidant activity and total phenol content in different flours.

	FOLIN (mg Acid Gallic/g Flour)			DPPH (mg Trolox/g Flour)		
Flour	Initial	G.D. *	I.D. **	Initial	G.D.	I.D.
BB	1.48 ± 0.01 ^b^	1.94 ± 0.05 ^c^	3.30 ± 0.06 ^a^	1.24 ± 0.04 ^b^	2.89± 0.53 ^b^	8.97 ± 0.73 ^d^
FBB	1.87 ± 0.24 ^b^	2.10 ± 0.00 ^d^	6.85 ± 0.03 ^c^	1.73 ± 0.09 ^c^	3.90± 2.21 ^d^	13.31 ± 1.63 ^f^
KB	1.59 ± 0.10 ^b^	1.82 ± 0.02 ^ab^	4.23 ± 0.09 ^a^	1.2 ± 0.01 ^b^	2.42 ± 0.51 ^a^	7.2 ± 0.29 ^c^
FKB	1.59 ± 0.01 ^b^	1.77 ± 0.06 ^a^	4.78 ± 0.20 ^b^	1.2 ± 0.08 ^b^	3.24 ± 0.04 ^c^	9.39 ± 0.01^e^
OG	0.85 ± 0.76 ^a^	1.88 ± 0.02 ^b^	2.72 ± 0.49 ^a^	0.40 ± 0.03 ^a^	2.27 ± 0.77 ^a^	3.04 ± 0.31 ^a^
FOG	2.89 ± 0.34 ^c^	2.12 ± 0.08 ^d^	4.91 ± 0.06 ^b^	1.30 ± 0.08 ^b^	2.85 ± 0.37 ^b^	5.04 ± 0.25 ^b^

* Gastric digestion; ** Intestinal digestion. Black bean (BB); black bean with *Pleurotus ostreatus* (FBB); kidney beans (KB); kidney bean with *Pleurotus ostreatus* (FKB); oats (OG) and oats with *Pleurotus ostreatus* (FOG). Values are the average of three replicates ± standard deviations, of three different lots. Mean values labeled with a different letter in the same column are significantly different (*p* < 0.05).

**Table 3:** Protein digestibility, soluble nitrogen and tannin content.

Flour	Protein Digestibility (%)	Soluble Nitrogen (%)	Tannin Content (mg/100 g)
BB	39.99 ± 1.71 ^a^	0.60 ± 0.80 ^b^	65.21 ± 0.027 ^f^
FBB	48.13 ± 0.78 ^c^	1.34 ± 2.3 ^d^	22.07 ± 0.016 ^a^
KB	44.06 ± 1.71 ^b^	0.30 ± 1.0 ^a^	35.54 ± 0.086 ^d^
FKB	69.01 ± 1.14 ^de^	0.60 ± 1.5 ^b^	23.37 ± 0.017 ^b^
OG	63.25 ± 1.65 ^d^	0.61 ± 0.4 ^b^	55.67 ± 0.057 ^e^
FOG	70.01 ± 0.30 ^e^	0.91 ± 0.22 ^c^	28.11 ± 0.030 ^c^

Black bean (BB); black bean with *Pleurotus ostreatus* (FBB); kidney beans (KB); kidney bean with *Pleurotus ostreatus* (FKB); oats (OG) and oats with *Pleurotus ostreatus* (FOG). Values are the average of three replicates ± standard deviations, of three different lots. Mean values labeled with a different letter in the same column are significantly different (*p* < 0.05).

**Table 4:** Effect of *Pleurotus ostreatus* on the amino acid profile.

Flours						
Amino Acid (mg/g Protein)	BB	FBB	KB	FKB	OG	FOG
Asparagine	125.29 ^e^	124.22 ^c^	125.40 ^e^	124.88 ^d^	87.30 ^a^	88.33 ^b^
Threonine	46.11 ^c^	47.30 ^d^	49.51 ^e^	49.56 ^e^	37.04 ^a^	39.17 ^b^
Serine	53.10 ^d^	51.60 ^c^	53.68 ^e^	52.96 ^d^	46.74 ^b^	45.00 ^a^
Glutamine	157.89 ^c^	154.80 ^b^	153.63 ^a^	154.52 ^b^	217.81 ^e^	212.50 ^d^
Proline	46.58 ^b^	48.73 ^c^	34.71 ^a^	49.08 ^d^	60.85 ^e^	61.67 ^f^
Glycine	41.92 ^a^	43.00 ^b^	44.89 ^c^	43.25 ^b^	54.67 ^d^	56.67 ^e^
Alanine	42.85 ^a^	45.87 ^d^	44.89 ^b^	45.19 ^c^	50.26 ^e^	53.33 ^f^
Valine	59.62 ^b^	64.50 ^f^	60.62 ^c^	63.65 ^e^	57.32 ^a^	61.67 ^d^
Methionine + Cysteine	20.44 ^a^	22.45 ^c^	21.29 ^a^	21.87 ^b^	47.62 ^d^	49.19 ^d^
Isoleucine	49.84 ^c^	53.03 ^d^	49.98 ^c^	52.96 ^d^	41.45 ^a^	45.83 ^b^
Leucine	87.10 ^d^	88.87 ^e^	86.07 ^c^	89.89 ^f^	82.01 ^a^	84.17 ^b^
Tyrosine	30.74 ^c^	32.97 ^e^	32.39 ^d^	33.04 ^e^	27.34 ^a^	28.33 ^b^
Phenylalanine	61.95 ^d^	63.07 ^e^	60.62 ^c^	63.65 ^f^	56.44 ^a^	57.50 ^b^
Hydroxylysine	1.40 ^a^	3.34 ^d^	1.39 ^a^	1.94 ^b^	2.65 ^c^	3.33 ^d^
Ornithine	0.47 ^a^	0.96 ^d^	0.93 ^c^	0.97 ^d^	0.88 ^b^	1.67 ^e^
Lysine	68.93 ^d^	52.56 ^b^	68.49 ^d^	56.37 ^c^	47.62 ^b^	34.17 ^a^
Histidine	28.88 ^f^	25.80 ^d^	28.69 ^e^	25.27 ^c^	22.93 ^b^	20.83 ^a^
Arginine	57.29 ^c^	49.21 ^a^	60.62 ^d^	51.02 ^b^	67.02 ^e^	60.83 ^d^
Tryptophan	10.71 ^c^	10.51 ^b^	11.11 ^d^	11.18 ^d^	14.99 ^e^	10.00 ^a^

Black bean (BB); black bean with *Pleurotus ostreatus* (FBB); kidney beans (KB); kidney bean with *Pleurotus ostreatus* (FKB); oats (OG) and oats with *Pleurotus ostreatus* (FOG). Mean values labeled with a different letter in the same file are significantly different (*p* < 0.05).
